# Experimental Porcine *Toxoplasma gondii* Infection as a Representative Model for Human Toxoplasmosis

**DOI:** 10.1155/2017/3260289

**Published:** 2017-08-13

**Authors:** Julia Nau, Silvia Kathrin Eller, Johannes Wenning, Katrin Henrike Spekker-Bosker, Horst Schroten, Christian Schwerk, Andrea Hotop, Uwe Groß, Walter Däubener

**Affiliations:** ^1^Institute of Medical Microbiology and Hospital Hygiene, Medical Faculty, Heinrich-Heine-University Düsseldorf, Universitätsstraße 1, 40225 Düsseldorf, Germany; ^2^Pediatric Infectious Diseases, Department of Pediatrics, Medical Faculty Mannheim, Heidelberg University, Theodor-Kutzer-Ufer 1-3, 68167 Mannheim, Germany; ^3^Institute for Medical Microbiology, University Medical Center, Kreuzbergring 57, 37077 Göttingen, Germany

## Abstract

Porcine infections are currently not the state-of-the-art model to study human diseases. Nevertheless, the course of human and porcine toxoplasmosis is much more comparable than that of human and murine toxoplasmosis. For example, severity of infection, transplacental transmission, and interferon-gamma-induced antiparasitic effector mechanisms are similar in pigs and humans. In addition, the severe immunosuppression during acute infection described in mice does not occur in the experimentally infected ones. Thus, we hypothesise that porcine *Toxoplasma gondii* infection data are more representative for human toxoplasmosis. We therefore suggest that the animal model chosen must be critically evaluated for its assignability to human diseases.

## 1. Introduction

*Toxoplasma gondii* (*T. gondii*) is one of the most prevalent parasites worldwide. This is due to the fact that *T. gondii* is able to chronically infect all warm-blooded animals including humans. Furthermore, its lifelong persistence in the host increases the chance of transmission. Definitive hosts are members of the *Felidae* family, which eventually shed environmentally resistant oocysts that are taken up by new intermediate or aberrant hosts (e.g., mice, pigs, or humans) via soil, food, or water [1, 2].

Since pork is the most frequently consumed meat in Europe, the *T. gondii* infection rate of pigs is of great interest [1, 3]. The prevalence of toxoplasmosis in pigs is commonly determined by the detection of anti-*T. gondii* antibodies in sera and meat juice. Detection rates of these porcine anti-*T. gondii* antibodies vary worldwide, ranging from 45% (*n* = 402) of pigs, analysed in Mexico [4], to 6.2%(*n* = 1368) positives in a seroprevalence study in Austria [5]. About 25% (*n* = 1200) positives were measured in Spain [6] and about 19% (*n* = 2004) in Germany [7].

A “meat juice serology” study, performed in Germany, demonstrated a prevalence of *T. gondii* of about 10% [8], whereas a prevalence of about 3% (*n* = 1549) was found in cardiac fluids in a French study [9]. Therefore, setting a benchmarking with the studies conducted so far is exceedingly difficult, since seroprevalence depends on the geographic region. The observed seroprevalence has also been influenced by other variables, such as the detection method, the age of the animals, the species, and the type of specimen. Moreover, prevalence studies performed within individual herds varied from 0 to 100%. Hence, the need for standardized test systems is growing.

One method to standardize serologic studies is the use of recombinant antigens as targets. *T. gondii* surface antigens (SAG) such as SAG1 [10] or secreted antigens have been successfully employed to analyse murine and porcine probes. Among the latter are the dense granule proteins (GRA proteins) which are secreted into the parasitophorous vacuole (PV) and integrated into the PV membrane (PVM) where they interact with host cell proteins and organelles [11]. For example, a GRA7-based enzyme-linked immunosorbent assay has emerged as a promising tool to study the prevalence of toxoplasmosis in pigs [12]. Furthermore, the important role of GRA antigens in immunity against *T. gondii* has also been underlined by the efficiency with which a GRA1-GRA7 DNA vaccine elicits cellular and humoral immune responses [13].

To summarize, several publications deal with the porcine humoral immune response after *T. gondii* infection, but data analysing cellular immune responses are relatively rare. Nevertheless, it is clear that toxoplasmosis initially results in the induction of a type 1 T helper cell (TH1) response in infected swine. Seroconversion was detected 20 days post infection (dpi) in orally infected mini pigs. An enhanced cellular immune response (indicated by increased CD25 expression on lymphocytes) was already detectable on day three postinfection, and significant IFN-*γ* production was detectable on day six postinfection [14]. Verhelst et al. described an upregulation of interferon response factor 1 (IRF1) and interferon-gamma (IFN-*γ*) gene expression in infected swine [12]. Dawson et al. were able to ascribe the elevated IFN-*γ* expression in *T. gondii*-infected pigs to peripheral blood mononuclear cells (PBMC) [15]. IFN-*γ* is the most prominent TH1 cytokine and known to be essential for a sufficient defence against *T. gondii* in different host species and cell types. Nevertheless, the effector mechanisms that are activated by IFN-*γ* in porcine cells have been unclear up to now.

Here, we show that infected pigs have a strong immune response against recombinant GRA antigens (rGRA), which is concomitant with a strong IFN-*γ* production by T cells mediating antiparasitic effects. We demonstrate that the growth control of *T. gondii* tachyzoites is mediated by the IFN-*γ*-induced expression of the tryptophan-degrading enzyme indoleamine 2,3-dioxygenase (IDO) in porcine choroid plexus epithelial (PCP-R) cells.

## 2. Materials and Methods

### 2.1. Infection of Pigs

All animal experiments were performed by Prof. A. M. Tenter (Institute for Parasitology, University of Veterinary Medicine, Hannover). The animal study, permit number DEC 2008.III.30.023, was reviewed and approved by the local animal ethics committee according to the recommendations of the EU directive 86/609/EEC. The number of animals used and their suffering was minimized. In a first study, sera were harvested from 10 orally infected animals (*T. gondii*; DX) and 7 uninfected controls. In the second part of this study, eight 7- to 9-week-old pigs were infected with *T. gondii* ME49 oocysts according to the protocol of Bokken et al. [16] and T cell responses were analysed. To simulate a natural infection, pigs were infected orally with 10^5^ oocysts in 2 ml drinking water per animal. Blood samples were taken at different time points (0, 7, 21, 28, 48, 98, and 159 days postinfection (dpi)). Heparinised blood samples were processed within 24 h and peripheral blood mononuclear cells (PBMC) were obtained by Ficoll gradient centrifugation and used immediately.

### 2.2. Production of Recombinant Antigens

The His-tagged recombinant antigens SAG1 and BAG1 were obtained from the Institute of Medical Microbiology of the University Medical Center Göttingen [17]. The recombinant antigens GRA1, GRA2, GRA7, and GRA9 were produced in the Institute of Medical Microbiology and Hospital Hygiene of the Heinrich Heine University in Düsseldorf as described previously [17]. All recombinant antigens were purified using a nickel-nitrilotriacetic acid (Ni-NTA) matrix under denaturating conditions and tested by SDS-PAGE, Coomassie blue staining, and Western blot analysis using anti-His antibody (QUIAGEN, Hilden, Germany). Protein concentrations were determined using a bicinchoninic acid (BCA) detection assay according to the manufacturer's instruction (Pierce, Rockford, IL).

### 2.3. Western Blot Analysis

For Western blot analysis, 10% NuPAGE Novex Bis-Tris Mini gels and the appropriate electrophoresis system from Invitrogen (Karlsruhe, Germany) were used. Recombinant proteins (2 or 6 *μ*g) were separated in the SDS-containing gels by electrophoresis for more than one hour at 160 V. Seeblue Plus2 marker was used as molecular mass standard. After the proteins were semidry blotted on nitrocellulose membranes (CarboGlas, Schleicher & Schüll, Dassel, Germany), membranes were blocked in 5% (*w*/*v*) skim milk powder in PBS for 1 h at room temperature. Then, the pig sera were used as primary antibody in a 1 : 100 dilution in 5% (*w*/*v*) skim milk powder in PBS. After incubation overnight at 4°C, membranes were washed three times in PBS containing 0.2% Tween for 5 minutes. Thereafter, membranes were incubated for 45 minutes at room temperature with goat anti-pig alkaline phosphatase-conjugated IgG (1 : 000 Dianova, Hamburg, Germany), diluted in 5% (*w*/*v*) skim milk powder in PBS. After three additional washing steps, bands were detected by the addition of substrate buffer for alkaline phosphatase (100 mM Tris-HCl, pH 9.5; 100 mM NaCl; and 10 mM MgCl_2_) and staining solution (33 *μ*l BCIP-T and 44 *μ*l NBT in 10 ml substrate buffer). The reaction was stopped by the addition of *aqua dest*. And membranes were laminated.

### 2.4. T Cell Proliferation Experiments

Peripheral blood mononuclear cells (PBMC) were prepared from the heparinised blood of infected and noninfected pigs after Ficoll density gradient centrifugation. For proliferation experiments, 1.5 × 10^5^ porcine PBMC per well were incubated in 200 *μ*l Iscove's modified Dulbecco's medium (IMDM; Gibco, Grand Island, USA) containing 5% foetal calf serum (FCS) and 1% PenStrep (Biochrom, Berlin, Germany).

Cells were stimulated with toxoplasma lysate antigen (TLA) (10^6^ lysed RH *T. gondii* parasites per ml), recombinant antigens (1 *μ*g/ml), concanavalin A (ConA; 1 *μ*g/ml), or left untreated as negative control. After three to five days of incubation (dependent on the result of microscopic examination), 0.2 mCi [^3^H]-thymidine was added for 24 h and T cell proliferation was determined using liquid scintillation spectrometry (1205 Betaplate, PerkinElmer, Jügesheim, Germany).

### 2.5. IFN-*γ* Assay

After a five-day stimulation period with TLA or recombinant antigens the amount of IFN-*γ* in supernatants of porcine PBMC cultures was determined using the porcine DuoSet ELISA (R&D Systems, Minnesota, USA) according to the manufacturer's instructions.

### 2.6. Culture and *In Vitro* Stimulation of PCP-R Cells

Porcine choroid plexus epithelial (PCP-R) cells were obtained from H. Schroten (Department of Pediatrics, Heidelberg University, Germany) [18] and cultured in IMDM containing 10% FCS, 0.05% insulin, and 1% PenStrep at 37°C in a 10% CO_2_-enriched atmosphere. Cultures were split 1 : 10 every 4 days using 0.05% trypsin-EDTA (Gibco).

1.5 × 10^5^ PCP-R cells/well were stimulated with different amounts of porcine IFN-*γ* (R&D Systems) for 72 h in 200 *μ*l cell culture medium or left untreated as negative control. Samples for subsequent kynurenine detection were supplemented with 100 *μ*g/ml L-tryptophan (Sigma Aldrich, St. Louis, USA). In addition, some samples were treated with 100 *μ*g/ml 1-L-methyl-tryptophan (1-MT; Sigma Aldrich), an IDO-specific inhibitor.

After three days of incubation, supernatants of stimulated or unstimulated PCP-R cells were harvested and the amount of kynurenine was determined using Ehrlich's reagent [19].

### 2.7. *T. gondii* Infection of PCP-R Cells

After 72 h of stimulation with or without IFN-*γ* in the presence or absence of 1-MT or additional L-tryptophan, PCP-R cells were infected with 10^5^* T. gondii* ME49 tachyzoites per well. The infected cells were incubated at 37°C in a 10% CO_2_-enriched atmosphere. After 24 h, 0.012 MBq [^3^H]-uracil was added to the cells, and after lysis of the cells, the cell culture plates were frozen at −20°C. *T. gondii* proliferation was determined using liquid scintillation spectrometry (1205 Betaplate, PerkinElmer, Jügesheim, Germany).

### 2.8. Statistical Analysis

All experiments were performed in triplicate, and data are given as mean ± standard error of a minimum of three independent experiments. For statistical analysis, the two-tailed unpaired *t*-test was used and significant differences are indicated with asterisks (^∗^*p* ≤ 0.05, ^∗∗^*p* ≤ 0.01, and ^∗∗∗^*p* ≤ 0.0001). Analysis was performed using GraphPad Prism software (GraphPad Software Inc., San Diego, CA).

## 3. Results

The oral uptake of *T. gondii* oocysts frequently results in an asymptomatic infection in pigs, which induces a robust production of parasite-specific antibodies. Therefore, seroprevalence studies are usually performed to detect *T. gondii* infection in pigs. In a first study, we therefore analysed the antibody production against recombinant *T. gondii* proteins in pigs infected orally with *T. gondii* (10^3^ or 10^5^ oocysts per animal). We performed Western blot analysis using recombinant *T. gondii* proteins GRA1, GRA2, GRA7, and GRA9. We found antibodies against GRA2, GRA7, and GRA9 98 dpi in all sera, irrespective of infection dose. However, antibodies against GRA1 were detected only infrequently (Table 1). No antibodies against *T. gondii* proteins could be detected in sera from uninfected animals (*n* = 7). This serologic data indicate that oral infection with 10^3^ as well as 10^5^ oocysts is effective.

**Figure 1:**
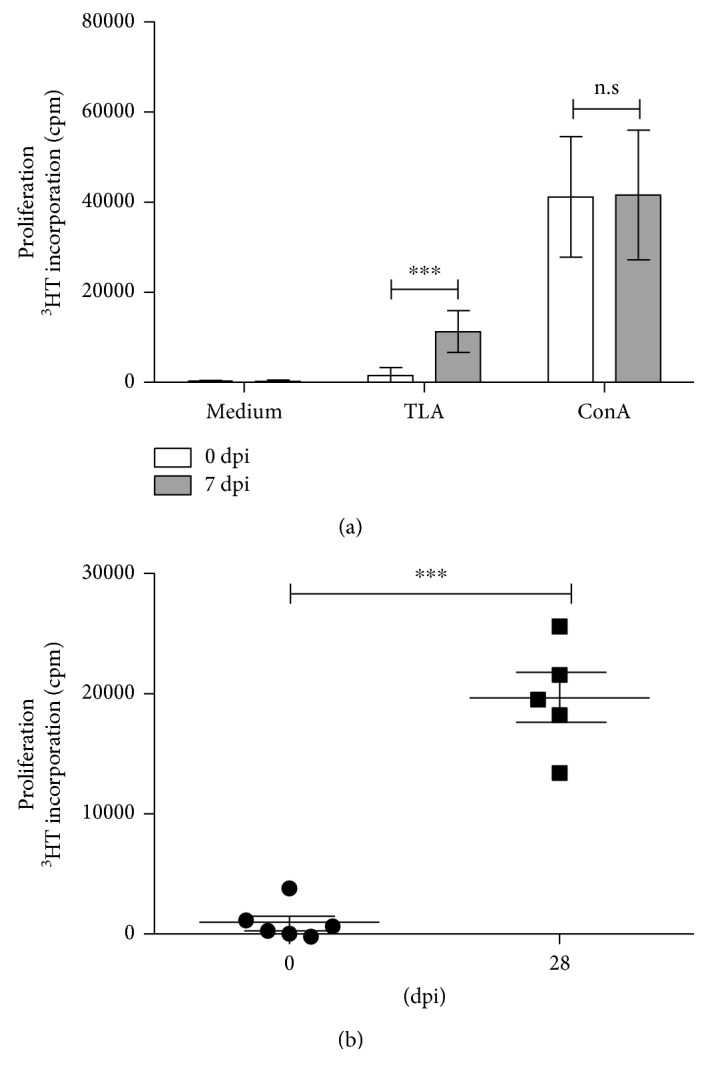
Toxoplasma antigen-specific T cell proliferation in cells from *T. gondii*-infected animals. (a) 1.5 × 10^5^ PBMC/well from 6 pigs before infection and 7 dpi were stimulated in triplicate with TLA or ConA for 4 days. Thereafter, ^3^H-thymidine was added for 18–24 h. Data are given as mean cpm ± SD from 6 individuals. (b) TLA-specific cell proliferation from uninfected and infected (28 dpi) animals. Each dot represents the result of one individual. Significant differences are marked with asterisks. Data are given as mean cpm ± SD from 5 individuals; (n.s. = not significant; ^∗∗∗^*p* ≤ 0.0001).

In the second part of our study, we focused on the cellular immune response during porcine toxoplasmosis. In the early phase of toxoplasmosis, a strong immunosuppression is described in mice [20, 21]. Hence, the ability of the parasite to interfere with the porcine immune system was analysed. To this end, the responsiveness of porcine T cells was determined prior to infection or at early time points postinfection (7 dpi). In these proliferation studies, peripheral blood mononuclear cells (PBMC) from infected and noninfected animals were stimulated with the T cell mitogen concanavalin A (ConA) or with TLA. After five days of incubation, T cell proliferation was determined by ^3^H-thymidine incorporation. As shown in Figure 1(a) T cells from noninfected and infected pigs respond comparably to the polyclonal stimulus ConA. Interestingly, only cells harvested from infected animals (7 dpi) show a significant proliferation after TLA stimulation. Comparable data were also obtained with cells harvested at 28 dpi shown in Figure 1(b). PBMCs, sampled 28 dpi and later (42, 56, 84, and 159 dpi) demonstrated a strong antigen-specific T cell proliferation already after 3 days of stimulation.

**Figure 2:**
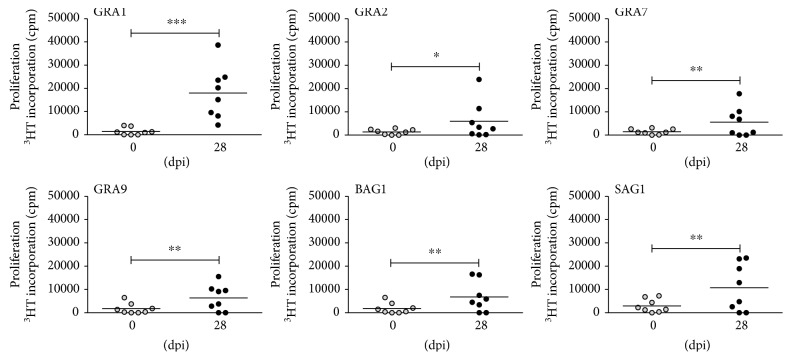
T cell proliferation after stimulation with recombinant antigens from *T. gondii*. 1.5 × 10^5^ PBMC/well from 8 pigs before infection (0 dpi) and day 28 post infection (28 dpi) were stimulated in triplicate with recombinant GRA1, GRA2, GRA7, GRA9, BAG1, or SAG1 (1 *μ*g/ml) and T cell proliferation was determined via ^3^H-thymidine incorporation. Each dot represents one individual; black bars indicate mean counts per minute from all individuals tested; significant differences are marked with asterisks (^∗^*p* ≤ 0.05, ^∗∗^*p* ≤ 0.01, and ^∗∗∗^*p* ≤ 0.0001).

In subsequent studies, the T cell response against several recombinant *T. gondii* antigens was analysed. In these experiments PBMC from infected pigs were stimulated with four different recombinant antigens (GRA1, GRA2, GRA7, and GRA9) at a concentration of 1 *μ*g/ml. In addition, recombinant SAG1 and BAG1 antigens were used since these are typical antigens characterising the tachyzoite and bradyzoite stage of *T. gondii*, respectively. Both antigens have previously been described as target antigens for murine and human T cells [21, 22]. After five days of stimulation with recombinant *T. gondii* proteins, T cell growth was monitored by ^3^H-thymidine incorporation. The data presented in Figure 2 were obtained with PBMC of 8 animals harvested 28 dpi and show that all recombinant *T. gondii* antigens tested can elicit a T cell response in infected animals, while no proliferative response was observed when PBMC from noninfected animals were stimulated with the same antigens. In this experiment, GRA1 was found to activate cells from five animals, GRA7, GRA9, and SAG1 were recognised by T cells from four animals, and T cells from two animals recognised only GRA2 or BAG1. However, every infected animal had T cells recognising at least one of the tested recombinant antigens. In further experiments, PBMC harvested 7, 28, and 48 dpi and were tested and comparable data were obtained.

**Figure 3:**
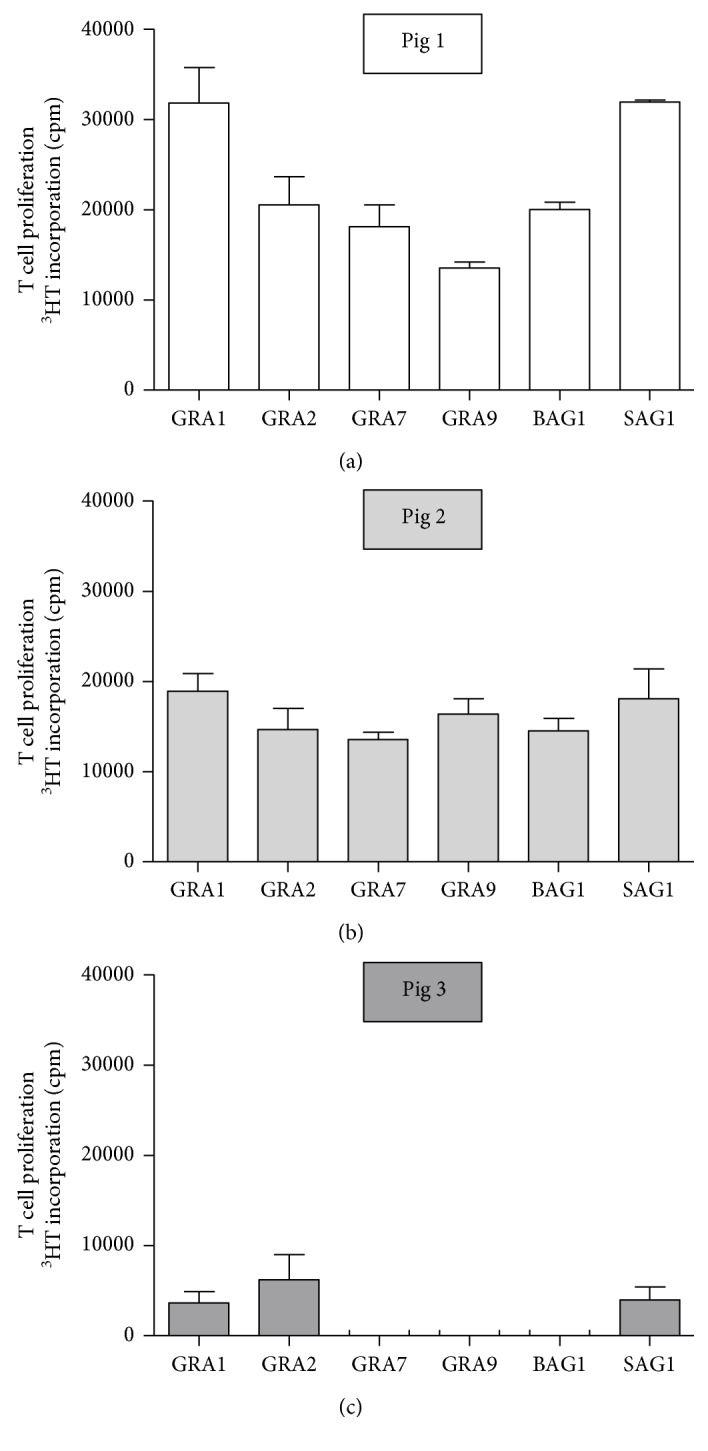
Differential response of PBMC harvested from different animals (pigs 1–3) to recombinant *T. gondii* antigens. 1.5 × 10^5^ PBMC harvested 7 dpi were stimulated with recombinant *T. gondii* antigens (1 *μ*g/ml). T cell proliferation was determined on day 5 after *in vitro* stimulation. Data are given as mean counts per minute (cpm).

Figure 3 depicts the individual *in vitro* reactivity of T cells from three different pigs after oral infection with oocysts (7 dpi). For example, pig number 1 developed a T cell response to all tested antigens with the strongest response directed against GRA1 and SAG1. Pig number 2 developed a comparable T cell response against all six antigens tested, while cells from pig number 3 showed a weak response to GRA1, GRA2, and SAG1 and no response was observed to GRA7, GRA9, and BAG1.

**Figure 4:**
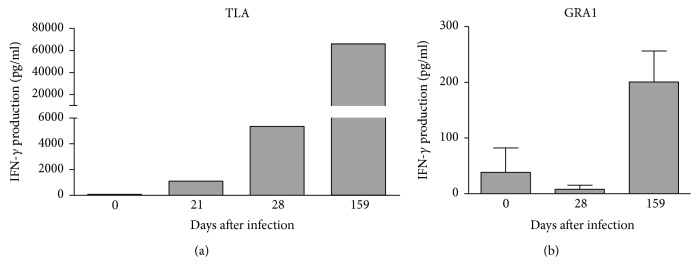
IFN-*γ* production by *T. gondii*-specific T cells. 1.5 × 10^5^ peripheral blood mononuclear cells from infected animals were stimulated with TLA (a) or GRA1 (b) for five days. Thereafter, supernatants were harvested and the IFN-*γ* concentration was determined by ELISA. Data are given as mean ± SD from two experiments, performed in duplicate (a) or as mean ± SD from four experiments, performed in duplicate (b).

In immunocompetent individuals, infection with *T. gondii* results in a robust stimulation of a TH1 response. Since IFN-*γ* is the most prominent TH1 cytokine, the IFN-*γ* production induced by stimulation with TLA (Figure 4(a)) or recombinant antigens (Figure 4(b)) was determined in PBMC from infected animals. IFN-*γ* is produced in high amounts by T cells harvested from blood after 28 dpi. A comparable IFN-*γ* production was also found in T cells harvested 159 dpi. Furthermore, T cells stimulated with recombinant antigens (e.g., GRA1) also produce IFN-*γ*, but the response is less intense as shown in Figure 4(b). Comparable data were also obtained with other recombinant antigens tested (data not shown).

IFN-*γ* induces a plethora of antimicrobial effector mechanisms. In previous studies, we discovered that native porcine PCP-R cells are able to restrict the growth of *Streptococcus suis* after IFN-*γ* stimulation. Furthermore, we showed that IFN-*γ* induced the expression of indoleamine 2,3-dioxygenase (IDO) and mediated antibacterial effects by tryptophan depletion [23].

**Figure 5:**
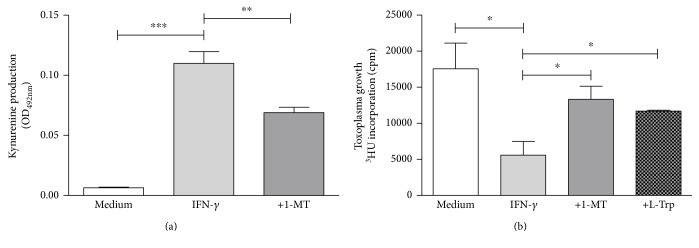
IFN-*γ* induces IDO activity and antiparasitic effects in porcine PCP-R cells. 3 × 10^5^ PCP-R cells were stimulated with IFN-*γ* (100 ng/ml). In some groups, the IDO inhibitor 1-methyltryptophan (1-MT) was supplemented (100 *μ*g/ml). Kynurenine concentration was determined in the supernatants using Ehrlich's reagent after three days of stimulation (a). Identical cell cultures were stimulated and afterwards infected with *T. gondii* (3 × 10^4^ ME49 tachyzoites per well). In the control group, L-tryptophan (L-Trp) (100 *μ*g/ml) was added at the time of infection. After three days, toxoplasma growth was monitored using ^3^H-uracil incorporation (b). Data are given as mean ± SD of triplicate cultures; significant differences were marked with asterisks (^∗^*p* ≤ 0.05, ^∗∗^*p* ≤ 0.01, and ^∗∗∗^*p* ≤ 0.0001).

Therefore, we tested whether IFN-*γ*-activated PCP-R cells [18] can restrict the growth of *T. gondii*. Data of a representative experiment are shown in Figure 5.

As expected, IDO is induced by IFN-*γ* in PCP-R cells. Interestingly, we found that these activated PCP-R cells can restrict the growth of *T. gondii*. Furthermore, this antiparasitic effect could be blocked either by tryptophan supplementation or by 1-MT (an IDO-specific inhibitor), indicating that IDO is involved in the defence of PCP-R cells against *T. gondii*.

The last experimental setting was designed to show whether the amount of IFN-*γ* produced by *T. gondii*-stimulated T cells is sufficient to reduce *T. gondii* proliferation in PCP-R cells. Therefore, T cell supernatants harvested from TLA-stimulated porcine T cells were added to PCP-R cells, and kynurenine production (indicating IDO activity) was determined after three days of culture.

**Figure 6:**
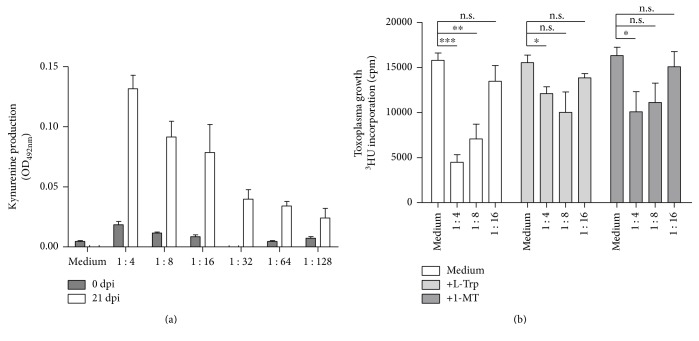
Supernatants of TLA-stimulated PBMC obtained from *T. gondii*-infected animals induce IDO activity and antiparasitic effects in porcine PCP-R cells. 3 × 10^5^ PCP-R cells were stimulated with serially diluted supernatants from TLA-activated porcine PBMC. (a) After three days of PBMC culture, supernatants were harvested and the kynurenine concentration was determined using Ehrlich's reagent. (b) Identical cell cultures were infected with *T. gondii* (3 × 10^4^ ME49 tachyzoites per well) after stimulation with T cell supernatants. In some cultures, L-tryptophan (100 *μ*g/ml) or 1 MT (100 *μ*g/ml) was added. After three days, toxoplasma growth was monitored using ^3^H-uracil incorporation. Data are given as mean ± SD of triplicate cultures; significant differences were marked with asterisks (n.s. = not significant; ^∗^*p* ≤ 0.05, ^∗∗^*p* ≤ 0.01, and ^∗∗∗^*p* ≤ 0.0001).

PCP-R cells stimulated with T cell supernatants harvested from culture supernatants from TLA-stimulated T cells (from *T. gondii*-infected animals) express IDO activity while supernatants harvested from cells from uninfected animals did not (Figure 6(a)). And, even more interestingly, supernatants harvested from TLA-stimulated PBMC from infected animals can induce a parasitostatic state in PCP-R cells which could be reversed by 1-MT or tryptophan supplementation (Figure 6(b)), indicating that the parasitostatic effect is based on IDO-mediated tryptophan degradation.

## 4. Discussion

The use of porcine cells to study human diseases is not very common. However, in the case of toxoplasmosis, the porcine model is superior to the murine model which is usually employed. For example, infection of pregnant pigs with toxoplasma results in abortion or in the birth of infected symptomatic and asymptomatic piglets [24, 25]. This resembles the human situation where an intrauterine infection might also lead either to abortion or to the birth of an infected child [1, 26]. The risk of mother to child transmission during primary toxoplasmosis depends on the gestation time. The transmission risk in the first trimenon is lower than in the third trimenon, and not all primary infections result in vertical transmission of the parasite [26], suggesting that a mechanism exists which protects the unborn child.

In contrast, abortion during toxoplasmosis in mice is mainly due to the abortogenic effect of the TH1 cytokine IFN-*γ* and infected pups are usually not found in immunocompetent mice [27, 28]. In addition, in humans and pigs, postnatally acquired toxoplasmosis in immunocompetent individuals usually only causes a mild clinical symptom (e.g., fever) or remains asymptomatic[29], while toxoplasmosis in inbred mice is, dependent on toxoplasma strain virulence and infection dose, a life-threatening disease. In contrast, *T. gondii* strains which are apathogenic in the murine system might cause clinical disease in humans and in pigs [30].

Usually, *T. gondii* infection in pigs results in the induction of a strong humoral and cellular immune response. Both are directed against soluble *T. gondii* proteins as well as surface antigens. The so-called “excreted secreted antigens” (ESA) from *T. gondii* have been found to be immunodominant antigens and consist of a mixture of GRA proteins from *T. gondii* including GRA1, GRA2, GRA7, and GRA9 [11].

When pigs are immunized with ESA preparations, they develop a strong cellular and humoral immune response resulting in resistance to infections with virulent type I strains. The porcine immune response has also been shown to be mainly directed against ESA proteins with molecular masses between 34 and 116 kDa. Furthermore, the immunization also results in reduced cyst formation in the muscle which might be beneficial for the consumer due to a reduced risk of infection [31].

We were therefore interested in analysing the humoral immune responses in sera of experimentally infected pigs directed against defined GRA proteins from *T. gondii*. Our Western blot analysis revealed that all orally infected animals developed a strong humoral immune response against GRA2, GRA7, and GRA9, while no antibodies directed against GRA1 were found. This is in accordance with published data from Verhelst et al. [12] who analysed the antibody production in pigs after oral infection with *T. gondii* tissue cysts. Using an ELISA technique, they found strong immunoreactivity to GRA7 and also no antibodies directed against GRA1. In addition, Jongert et al. immunized pigs with a GRA1-GRA7 cocktail DNA vaccine [13]. After immunization with this DNA vaccine, they found a strong humoral immune response directed against GRA7 and GRA1, whereas no detectable immune response was elicited in animals vaccinated with tachyzoites of the RH strain. However, RH-vaccinated animals developed high amounts of anti-GRA7 antibodies, but not anti-GRA1 antibodies after reinfection with *T. gondii* tissue cysts. Altogether, we confirmed previous findings concerning the immunogenicity of GRA1 and GRA7 and additionally demonstrated a high immunogenicity of GRA2 and GRA9.

Antibodies in combination with complement can only kill extracellular parasites; therefore, a cellular immune response is necessary to control intracellular *T. gondii*. However, the strong antigen-specific T cell response of porcine cells even in the acute phase of toxoplasmosis (7 dpi) stands in striking contrast to data published with murine cells. Murine spleen cells harvested within the first weeks after *T. gondii* infection are unable to respond to stimulation with ConA or TLA. Detailed analysis showed T cell suppression in spleen cells from *T. gondii*-infected mice. This suppression is mainly due to an IFN-*γ*-dependent induction of the inducible nitric oxide synthase (iNOS) enzyme activity [20, 32].

In contrast, peripheral blood lymphocytes from patients with acute, symptomatic toxoplasmosis can mount a proliferative response after stimulation with soluble toxoplasma antigen [33]. We also found strong toxoplasma antigen-specific proliferation after stimulating PBLs from three different donors with acute symptomatic toxoplasmosis (own unpublished observations). Therefore, T cell reactivity during acute toxoplasmosis is comparable between humans and pigs but not mice.

Activated T cells usually do not interact directly with *T. gondii*, but T cell cytokines can induce antiparasitic mechanisms in different cell types. Several publications confirm that pigs, like many other species including humans, mount a TH1-type immune response during toxoplasmosis. Ex vivo analysis showed enhanced proliferation and expression of activation markers on T cells and increased IFN-*γ* production by T cells from infected animals [15, 31]. Furthermore, increased amounts of IFN-*γ* in sera of infected pigs were described as well as increased transcription of IFN-*γ* and IRF-1 [12, 14].

Our results support these findings since we found a strong proliferative T cell response after stimulation of PBMC with TLA in all animals orally infected with *T. gondii*. In addition, these T cells produced high amounts of IFN-*γ*. However, only little information is available concerning T cell stimulation with defined *T. gondii* antigens. For example, Jongert et al. saw that the GRA1-GRA7 DNA vaccine-immunized pigs developed GRA1- and GRA7-specific T cells and that these T cells produced the TH1 cytokine IFN-*γ* [13]. Here, we prove that all tested recombinant GRA and surface antigens were recognized by T cells of infected animals. However, GRA1, which causes no or only a weak humoral immune response, initiates strong T cell activation. Furthermore, we found that GRA-specific T cells can produce IFN-*γ*. However, the amount of IFN-*γ* produced in comparison to that of TLA-stimulated cells was low. This might be due to polyclonal T cell stimulation via the complex toxoplasma lysate antigen in contrast to the mono- or oligoclonal stimulation via defined recombinant antigens.

IFN-*γ* is well known as potent inducer of antimicrobial effector mechanisms; among them, the inducible nitrite oxide synthase (iNOS) [34], interferon-induced GTPases [35], and tryptophan-degrading enzyme IDO [36] are most important in the defense against *T. gondii* in several species. For example, iNOS was frequently reported to inhibit the growth of parasites *in vitro* and *in vivo* especially in mice. However, despite the observation that nitric oxide is involved in the pathophysiological processes during septic shock and LPS response in pigs [37, 38] and that iNOS is upregulated in the central nervous system of swine following infection with pseudorabies [39], iNOS is not an essential part of the innate immune response in pigs [40].

Interferon-induced GTPases were described to be involved in antimicrobial defence. Among them, Mx proteins, mainly dependent on type 1 interferon induction, mediate antiviral effects in human, murine, and porcine cells [41, 42].

Additionally, p47 GTPases mainly stimulated by IFN-*γ* were described to mediate antimicrobial effects in murine [42] but not in human cells [43], while data on porcine cells are lacking. The third family of IFN-induced GTPases is the guanylate-binding proteins (GBPs). Different mGBPs can mediate antitoxoplasma effects in murine cells [44, 45]. More recently, human GBP-1 was described to mediate antitoxoplasma effects in human mesenchymal stem cells [46]. No data concerning GBP-mediated antiparasitic effects have been published with porcine cells up to now; however, very recently, GBP1 was found to be a mediator of antiviral effects in a porcine kidney cell line [47].

The IFN-*γ* induced induction of IDO with subsequent degradation of tryptophan and inhibition of toxoplasma growth was first recognized by Pfefferkorn in 1984 [36]. Meanwhile, many human cells were found to use this mechanism to inhibit the growth of *T. gondii* tachyzoites and other tryptophan-auxotroph microorganisms [48]. Overall, only few papers have been published showing IDO-mediated effects in porcine cells. For example, porcine IDO was cloned in 2012 and was found to be more similar to human IDO than murine IDO [49]. On a functional level, porcine IDO was linked to protective effects in transplant arteriosclerosis and in xenoreactions [50, 51]. Furthermore, the role of IDO in the porcine lung in inflammation models has been well studied and IDO-mediated effects against *Streptococcus suis* have been described [23, 52].

In this manuscript, we show for the first time that the induction of IDO is responsible for the inhibition of *T. gondii* growth in porcine cells. We found that PCP-R cells can restrict the growth of *T. gondii* after stimulation with IFN-*γ* or after coincubation with T cell supernatants harvested from TLA-stimulated cells originating from infected animals. This antiparasitic effect could be blocked, at least in part, by addition of the IDO-specific inhibitor 1-MT or by supplementation of large amounts of tryptophan. IDO- and iNOS-mediated antiparasitic effects were found to be species-specific. Furthermore, we found that in porcine and human cells, IDO induction is the most important antiparasitic effector mechanism directed against *T. gondii*. Thus, antimicrobial effects in human and porcine cells were similar, but different from those in murine cells [53, 54].

IDO and iNOS mediate antimicrobial and immunoregulatory effects and human as well as porcine mesenchymal stem cells can inhibit alloantigen-driven T cell responses [55, 56]. Interestingly, also in respect to immunoregulatory effects, a species-specific difference is described. For example, in human and porcine mesenchymal stem cells, IDO was found to mediate immunosuppressive effects, while iNOS was involved in immunoregulation mediated by murine MSC [57]. Once again, porcine cells act more like human cells than murine cells do.

## Figures and Tables

**Table 1:** Immunoreactivity of sera from *T. gondii*-infected pigs to different recombinant GRA antigens (GRA) based on Western blot analyses.

Frequency of positive detection (%)				
	GRA1	GRA2	GRA7	GRA9
Sera of uninfected pigs (*n* = 7)	0	0	0	0
Sera of infected pigs 98 dpi with 10^3^ oocysts (*n* = 7)	20	100	100	80
Sera of infected pigs 98 dpi with 10^5^ oocysts (*n* = 7)	0	100	100	100
